# Spina Bifida

**Published:** 1902-09-13

**Authors:** 


### SPINA BIFIDA.

In the days before antiseptics spina bifida was a malformation for which surgery offered no very hopeful prospect of cure. Now things are on a very different footing. How great has been the change was well shown by the remarks made by Mr. Harold Styles at Manchester. He says that the treatment of this affection has now been placed upon the same rational footing as that of herniæ elsewhere, and that the radical operation of excision of the sac should take the place of all other methods. Simple tapping should never be resorted to as a curative procedure, as it is followed by rapid accumulation of the fluid, and if frequently repeated there is apt to be leakage which leads sooner or later to death from meningitis or exhaustion; indeed this measure is only valuable when employed as a palliative, where the tumour is tense and ulcerated, and there is hope that by withdrawing the fluid perforation may be avoided and time be given for the ulcer to heal before proceeding to excision. When so used leakage may generally be prevented by employing a comparatively fine hollow needle, by passing this obliquely through the muscles into the sac, and by the application of a collodion dressing. Apart from excision, injection with Morton's fluid is the only treatment worth considering, and it cannot be denied that this method has been the means of curing many cases. But it is attended with greater danger than aseptic excision. Moreover, the cases in which injection with Morton's fluid is least dangerous are just those where excision is almost uniformly successful. In searching for the factors which should influence us in selecting or rejecting cases for operation, Mr. Styles says that if we hold the view that congenital hydrocephalus is incurable the co-existence of that affection constitutes a definite bar to operation. Then again if paralysis be due to imperfect development of the nerve elements operation cannot be expected to have any beneficial effect where it is present, and to attempt to preserve life under such conditions would be the reverse of humane. But there is reason to believe that in some cases a certain degree of paralysis may be due to tension of the nerve elements, and this being so, Mr. Styles says that he would be inclined to operate on cases with partial paralysis, but to reject those in which there is complete paralysis of the lower extremities and sphincters, particularly if talipes exists as well. Operation is also contraindicated when the base of the tumour is so large and the skin over it so thin that it is impossible to get a sufficiently sound covering for the wound. As to age it is better to wait until the child is a few months old, but if the tumour is enlarging rapidly, and is threatening to ulcerate or perforate, the operation must be performed no matter how young the child may be.

